# Hepatitis C: viral and host factors associated with non-response to pegylated interferon plus ribavirin

**DOI:** 10.1111/j.1478-3231.2010.02283.x

**Published:** 2010-10

**Authors:** Tarik Asselah, Emilie Estrabaud, Ivan Bieche, Martine Lapalus, Simon De Muynck, Michel Vidaud, David Saadoun, Vassili Soumelis, Patrick Marcellin

**Affiliations:** 1 INSERMU773, Centre de Recherche Bichat-Beaujon CRB3, Paris, France; 2 Service d'hépatologie, Hôpital BeaujonClichy, France; 3 INSERMU745, Université René Descartes, Paris, France; 4 Service de Biochimie, Hôpital BeaujonClichy, France; 5 Service de Médecine Interne, Hôpital Pitié-SalpétrièreParis, France; 6 INSERM U653, Institut CurieParis, France

**Keywords:** antiviral, boceprevir, immunity, interferon-stimulated genes, pegylated-interferon, polymerase inhibitors, protease inhibitors, STAT-C, sustained virological response, telaprevir

## Abstract

Treatment for chronic hepatitis C virus (HCV) infection has evolved considerably in the last years. The standard of care (SOC) for HCV infection consists in the combination of pegylated interferon (PEG-IFN) plus ribavirin. However, it only induces a sustained virological response (SVR) in half of genotype 1-infected patients. Several viral and host factors have been associated with non-response: steatosis, obesity, insulin resistance, age, male sex, ethnicity and genotypes. Many studies have demonstrated that in non-responders, some interferon-stimulated genes were upregulated before treatment. Those findings associated to clinical, biochemical and histological data may help detect responders before starting any treatment. This is a very important issue because the standard treatment is physically and economically demanding. The future of HCV treatment would probably consist in the addition of specifically targeted antiviral therapy for HCV such as protease and/or polymerase inhibitors to the SOC. In genotype 1 patients, very promising results have been reported when the protease inhibitor telaprevir or boceprevir is added to the SOC. It increases the SVR rates from approximately 50% (PEG-IFN plus ribavirin) to 70% (for patients treated with a combination of PEG-IFN plus ribavirin plus telaprevir). Different elements are associated with non-response: (i) viral factors, (ii) host factors and (iii) molecular mechanisms induced by HCV proteins to inhibit the IFN signalling pathway. The goal of this review is to present the mechanisms of non-response, to overcome it and to identify factors that can help to predict the response to anti-HCV therapy.

Hepatitis C virus (HCV) is among the leading causes of chronic liver disease worldwide and affects approximately 170 million people (1–3). HCV has been identified in 1989 as an enveloped virus with a 9.6 kb single-stranded RNA genome, member of the *Flaviridae* family, genus *Hepacivirus* (4–8). Six genotypes of HCV (from 1 to 6) and various subtypes have been identified (5). The severity of the disease associated with HCV infection varies from asymptomatic chronic infection to cirrhosis and hepatocellular carcinoma (1, 9).

Treatment of HCV using combination of pegylated interferon (PEG-IFN) plus ribavirin fails in about 50% of the patients and is physically and economically demanding. Thus, it is highly important to understand the mechanisms of non-response to overcome it and to identify factors that can help to predict the chance of each patient to respond to the treatment. Different elements are associated with non-response: (i) viral factors, (ii) host factors and (iii) molecular mechanisms induced by HCV proteins to inhibit the IFN signalling pathway. The goal of this review is to present the different factors associated with non-response to the current treatment against HCV (Fig. 1).

**Fig. 1:**
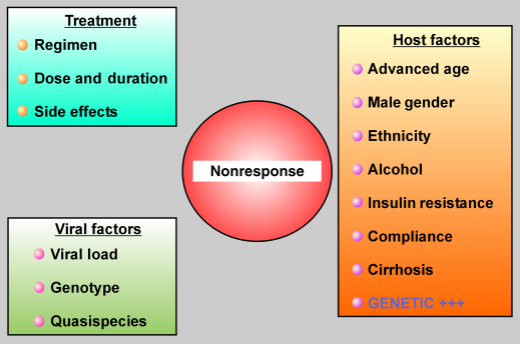
Factors associated to non-response to pegylated interferon plus ribavirin treatment.

## Activation of interferon pathway

Interferon type 1 are the major antiviral cytokines. HCV infection may induce host signalling pathways leading to IFN secretion (10–12). dsRNA viruses are known to induce IFN signalling pathways; the double-stranded RNA is recognized by cellular pattern recognition receptor such as TLR3 and RIG-I.

Although HCV is a single-stranded RNA virus, its replication may produce some dsRNA because of its RNA-dependent RNA polymerase NS5B. This dsRNA may activate the IFN signalling pathway (13).

The activation of TLR3 after the binding of dsRNA activates a cascade of events. IRF3 is phosphorylated and transcription factors such as NFκB and AP-1 are activated. Phosphorylated IRF3 forms a dimer and translocates into the nucleus where it binds to DNA to regulate the expression of IFNβ.

Receptors such as RIG-I and Mda5 recruit the IFNβ promoter stimulator 1 (IPS-1 or cardif) after the binding of dsRNA (10). IPS-1 plays an important role in the activation of IRF3, IRF7 and NFκB. IRF-7 forms a dimer and translocates into the nucleus to induce IFN α/β.

IRF-3 dimers collaborate with NFκB also to induce IFN α/β.

Interferon α/β binds to a receptor at the cell surface, inducing the activation of the Jak/STAT signalling pathway. In collaboration with IRF-9 and ISGF3, Jak/STAT signalling induces the activation of IFN-stimulated response elements activating the transcription of IFN α/β-stimulated genes (12).

**Fig. 2:**
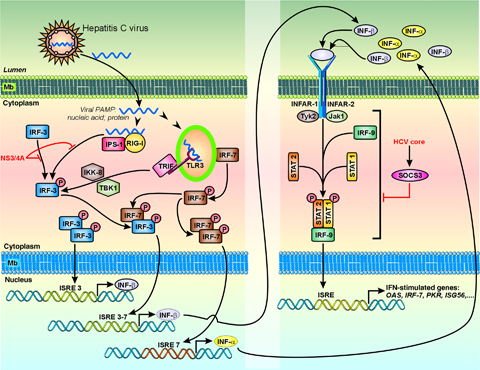
Hepatitis C virus (HCV) and immune response. Activation of toll like receptor 3 (TLR3) leads to the recruitment of IκB kinase (IKK)-related kinases, TANK-binding kinase 1 (TBK1) and IKKi. These kinases, together with adaptators TANK and NAP1, catalyse the phosphorylation of interferon (IFN) stimulatory factor-3 (IRF-3). Phosphorylated IRF-3 forms a dimer, translocates into the nucleus, binds to DNA in collaboration with transcription factor AP-1 and NF-κB and regulates the expression of IFNβ. The HCV NS3-4A serine protease may block the phosphorylation and effector action of IRF-3. After recognition of viral RNA, RIG-I and Mda5 recruit IFNβ promoter stimulator-1 (IPS-1) via caspase recruitment domain (CARD-CARD) interaction. IPS-1 is localized in the mitochondria and acts as an adaptator that plays a critical role in the activation of IRF-3 and IRF-7. IPS-1 is targeted and inactivated by the serine protease NS3/4A from HCV. IRF-7 forms a dimmer and translocates into the nucleus to induce IFN α/β. Endogenous IFN α/β bind to a common receptor (IFNAR-1/2) expressed at the cell surface of target cells. Receptor engagement leads to recruitment of Tyk2 and Jak1. Together with IRF-9 the two kinases induce activation of STAT1 and STAT2 which, together with ISGF3G/IRF9, bind to cis-acting IFN stimulated elements (ISREs), thereby activating the transcription of IFN α/β-inducible genes such as PKR, IL-8, OAS. The HCV core protein has been shown to induce the expression of SOCS3 (suppressor of cytokine signalling 3), which can suppress Jak/STAT.

This finally results in the production of proteins such as RNAse L and protein kinase R that will target the degradation of viral RNAs and block their translation (14) (Fig. 2).

## Definition of non-response

The standard of care (SOC) consists in the combination of PEG-IFN with ribavirin (15–19). The duration of the treatment can vary depending on the genotype. Forty-eight weeks are recommended for patients infected with genotypes 1 and 4, while patients with genotypes 2 and 3 are treated only for 24 weeks.

The goal of such a treatment is to obtain a sustained virological response (SVR) defined as undetectable HCV RNA in the serum after 24 weeks of post-treatment follow-up (15). This SVR results in the eradication of HCV infection and improvement of the histological outcome (20).

**Fig. 3:**
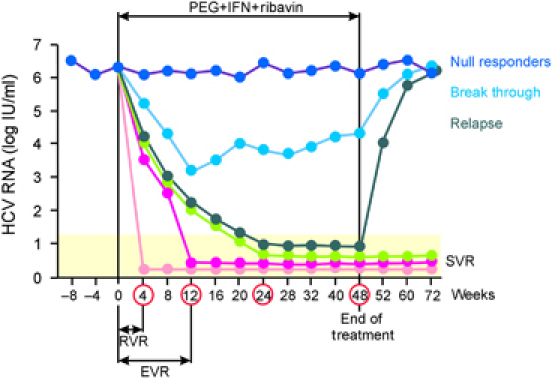
Definition of response to pegylated interferon (PEG-IFN) plus ribavirin treatment. The kinetic of HCV RNA level during PEG-IFN plus ribavirin therapy helps to predict the response. Rapid virological response (RVR) is defined as a HCV RNA negative at treatment week 4. Early virological response (EVR) consists in a reduction of HCV RNA >2 log compare to HCV RNA baseline, at week 12. A complete EVR is defined as a negative HCV RNA at the end of the 24 or 48 weeks of the treatment. Sustained virological response (SVR) is characterized by an absence of HCV RNA, up to 24 weeks after cessation of treatment. Reappearance of HCV RNA in the serum during the treatment is defined as a breakthrough. The term of relapse is used when HCV RNA level is again detectable when the therapy is discontinued. Finally, patients who do not respond to the treatment are described as (i) non-responders when they fail to clear HCV RNA after 24 weeks of therapy, or (ii) Null responders when they fail to clear the HCV RNA level up to 2 log at week 24 of the therapy, or (iii) Partial responders when they achieved a EVR and show HCV RNA level still detectable at week 24 of the therapy.

During the whole period of the treatment, HCV RNA load is monitored. Flat non-responders show no variation of the HCV RNA. Patients achieving an early virological response (more than 2 log10 reduction in HCV RNA load compared with the baseline or HCV RNA negative, at week 12) or a rapid virological response (RVR) (HCV RNA negative at treatment week 4) have a better chance of maintaining an SVR (15, 21–25) (Fig. 3).

Although a significant percentage of patients with chronic hepatitis genotypes 1 and 4 require 48 weeks of therapy, those with RVR might be treated for 24 weeks.

## Viral factors responsible for non-response to interferon treatment

### Genotype

Hepatitis C virus genotype is the strongest baseline predictor of IFN response. So far, 6 genotypes (1–6) have been described based on a sequence divergence. In the same genotype group, variants share at least 70% of sequence homology. Genotype 1 (subtypes 1a and 1b) is the most common in Europe, followed by genotypes 2 and 3. Genotypes 4–6 are less common, but are starting to be observed more frequently because of increasing cultural diversity.

The actual SOC therapy, in a patient with hepatitis C, yields a sustained response in approximately 55% of the cases. In patients with HCV genotypes 2 or 3, the SVR rates reaches 80%, while with genotype 1, patients SVR rates is only 50%. Finally, response rates for genotype 4 are higher than those for genotype 1, but lower than those for genotype 3 (approximately 65%).

## Genetic diversity

During its replication, HCV has the particularity to generate some quasispecies. Two quasispecies are characterized by at least 90% of nucleotide sequence homology (20, 26, 27). Thus, sensitivity to HCV therapy can be variable, because in this 10% of genetic divergence, it is possible to generate some viral variants with a different sensibility to the treatment.

In a recent study (28), the authors analysed the amino acid sequence of about 100 patients before treatment with IFN plus ribavirin. They show that the HCV sequence of non-responders presents three-fold more hydrophobic pairs of amino acids than the sequence of responders. These hydrophobic amino acids were predicted to contribute to IFN treatment failure by stabilizing the HCV proteins complex. The authors report that, using these algorithms, they were able to predict response to the treatment in 95% of the patients. Although these results are promising, they need to be confirmed in further studies.

Mutation in several subgenomic regions of HCV have been related to sensitivity to IFN treatment. Two amino acid regions of NS5A have been described and are thought to play a role in response to IFN treatment: (i) IFN sensitivity-determining region (29, 30) (ii) IFN/ribavirin resistance-determining region (IRRDR) (31). In both cases, the authors showed that the high number of mutations within these amino acid sequences (more than six in the case of IRRDR) is significantly associated with higher rates of SVR. However, these results need confirmation; other studies in other population of patients have not confirmed these data (32). Mutations within E2-PePHD, NS5A-PKRBD and NS5A-V3 have been reported later (33, 34). These mutations have been implicated in influencing the response to IFN therapy.

## Baseline viral load

Interestingly, several studies have demonstrated that the chance to respond to IFN treatment is related to the baseline viral load. Patients with a high viral load >800 000 UI/ml (2 millions of copies/ml) are less sensitive to the treatment than patients with a low viral load <800 000 UI/ml (21–24, 35–37). Thus, patients with genotype 1, low baseline viral load and RVR may be treated for 24 weeks, while patients with genotype 3, high baseline viral load and without RVR may require 48 weeks of treatment.

## Viral kinetics

Although PEG-IFN plus ribavirin induced an SVR in 80% of patients infected by genotype 2 or 3, only 50% of the patients with genotype 1 present an SVR.

When response rates are low (genotype 1 and 4), kinetics of the virus during the first weeks of the treatment are valuable indicators. The presence of an RVR, EVR or no significant decrease of viral load helps to predict the chance of achieving an SVR.

Patients who present an RVR have a greater chance of achieving an SVR (higher than 85%) (25). Patients who do not present any decrease of the viral load will not be able to respond to the treatment (35, 38–40).

## Host factor responsible of non-response to interferon treatment

Responsiveness to hepatitis C virus therapy depends not only on viral factors but also on host factors. Age, gender, cirrhosis, steatosis, insulin resistance, diabetes, African American ethnicity and weight (BMI) are all events associated to poor response to pegylated-IFN plus ribavirin treatment. Insulin resistance, obesity and steatosis are also associated with a higher risk of fibrosis progression (41, 42). The adherence of each patient is highly variable. Chances of achieving an SVR significantly decrease when patients receive <80% of the total dose of peg-IFN and/or <80% of total ribavirin and/or during <80% of the total period of treatment (43).

Comorbidities such as HIV and/or HBV co-infection, excess alcohol intake and drug use are generally associated with lower SVR rates (44). It seems that cannabis receptor stimulation is associated with lower response to IFN treatment (45). However an interesting study showed that occasional users of cannabis should not stop their consumption because at a low –dose, cannabis does not significantly inhibit IFN treatment but should reduce their observance.

Moreover, it has been recently reported that patients with a history of depression who were not receiving antidepressants and active intravenous drug users are more likely to fail treatment for genotype 2/3 HCV and will need additional support (46).

Insulin resistance has been shown to reduce the chances of achieving an SVR (47, 48). Both impaired fasting glucose and type-2 diabete mellitus are associated with lower rates of SVR in patients treated with peg-IFN and ribavirin. The use of insulin-sensitizing agents such as pioglitazone to HCV treatment increases both SVR and RVR rates (49). HCV expression induces insulin resistance (47). The core protein, through activation of the Januse Kinase pathway, inhibits the activity of insulin receptor 1 (IRS1) (50, 51). Moreover, expression of the core protein induces the activation of the SOCS-3 protein, which increased the degradation of both IRS1 and 2 (52).

In 2006, the protein USP18 has been reported to modify pharmacokinetic of IFN. USP18 is thought to inhibit the effect of IFN therapy by reducing its disponibility. Interestingly, a high level of USP18 expression has been associated with non-response to IFN treatment (53). Therapy targeting USP18 could be used in the future to limit its inhibitory effect and thus to increase SVR rates.

## Identification of new molecular markers related to non-response

Because of the important side effects and high cost of PEG-IFN plus ribavirin therapy, it is highly important to identify some molecular markers that can discriminate patients who will not respond to the treatment.

### Single nucleotide polymorphism, genome wide association studies

Large-scale and genome-wide association studies have attempted to identify a molecular pattern associated with responsiveness to IFN treatment. An SNP is a modification of one nucleotide of the genome sequence. It can occur in a coding region or in a non-coding region as well. Thus, in some particular cases, it exerts a direct effect on protein activity or expression.

Lin *et al.* (54) used an artificial neural network to address this kind of interaction in the antiviral treatment response for 523 patients with chronic hepatitis C. These patients had received IFN plus ribavirin treatment; 350 were sustained responders, while 173 were non-responders. They focused on 6 candidates genes involved in the IFN pathway (*ADAR, CASP5, ISCBP1, IFI44, PIK3CG* and *TAP2*). A total of 20 single nucleotide polymorphisms (SNPs) have been selected. The results of this study strongly support the notion that viral genotype and IFI44, members of interferon-stimulated genes (ISGs), play a role in the pharmacodynamic of IFN treatment (54).

Another study, by Huang *et al.* (55) in 2007, identified eight SNPs, spanning the entire IFN-γ gene in two different cohorts. The authors assessed the association between those polymorphisms and treatment response or clearance of the virus. Interestingly, an SNP variant located in the proximal IFN-γ promoter region next to the binding region of the heat shock transcription factor (HSF) C764G was significantly associated with SVR. Functional analyses show that the G allele confers a higher promoter activity and a stronger binding affinity for HSF1. The study suggests that the IFN-γ promoter SNP C764G is functionally important in determining viral clearance and treatment response to IFN (55).

Gene expression in liver biopsies and/or serum from responders and non-responders has been compared. Hwang *et al.* (56) isolated seven genes associated to responsiveness (*ADAR, CASP5, FGF1, ICSBP1, IFI44, TAP2* and *TGFBRAP1*). These genes have been used to construct a signature model by multiple logistic regression. The sensitivity was only 68% and the specificity was 60%. The model did not investigate gene–gene and gene–environment interactions (56) (Table 1).

**Table 1:** Host genetic diversity and response to interferon

Authors	Year	Technology	Number of patients	Identified targets
*Genome-wide association studies*				
Hwang *et al.* (56)	2006		217 (195 R and 122 NR)	26 SNPs in seven genes: *ADAR, CASP5, FGF1, ISCBP1, IFI44, TAP2, TGFBRAP1*
Lin *et al.* (54)	2006	DNA extraction from whole blood, polymorphism genotyping and artificial neural network	523	20 SNPs in six genes *ADAR, CASP5, ISCBP1, IFI44, PK3CG and TAP-2*
Huang *et al.* (55)	2007	DNA extraction from whole blood, polymorphism genotyping	284	Eight SNPs in IFN-γ
				IFN-γ 764G/C is associated with IFN response
Ge *et al.* (57)	2009	Blood	1137	Upstream *IL-28B*
Tanaka *et al.* (59)	2009	Blood	154+72	Upstream *IL-28B*
Suppiah *et al.* (58)	2009	Blood	555	Upstream *IL-28B*
Rauch *et al.* (60)	2010	Blood	1362	Upstream *IL-28B*
Asselah *et al.* (62)	2010	Blood	832	Upstream *IL-28B*

*Gene expression studies (transcriptome)*				
Chen *et al.* (66)	2005	mRNA extraction and RT-PCR on liver biopsies	51 (15 NR, 16 R, 20 control)	18 interferon-stimulated genes (ISGs) including *ISG15* and *USP18*
Asselah *et al.* (67)	2008	mRNA extraction and RT-PCR on liver biospies	69	*IFI-6-16, IFI27, ISG15, MX1, HERC15, TGFB2, OAS2, VEGFD, IL8, IFIT1*
Younossi *et al.* (70)	2009	mRNA extraction and RT-PCR on PBMC	68	*STAT6*, suppressor of cytokine signalling 1
Thomas *et al.* (61)	2009	Blood, genotype cohorts for rs12979860 3 kb upstream IL-28B	388+620	Upstream *IL-28B*
Sarasin-Filipowicz *et al.* (80)	2009	Liver biopsies	42	Decreased miR-122 in non-responders

*Studies performed on serum*				
Butera *et al.* (68)	2005	Immuno-quatification in plasma samples	58	*CXCL9* and *CXCL10* decrease and *CXCL11*=after effective antiviral therapy
Paradis *et al.* (71)	2006	Surface-enhanced laser desorption ionization time-of-flight mass spectrometry on serum samples	96	37 protein peaks in responders and two in non-responders
Younossi *et al.* (70)	2009	mRNA extraction and RT-PCR on PBMC	68	*STAT6*, suppressor of cytokine signalling 1

IFN, interferon; IL, interleukin; RT-PCR, real-time polymerase chain reaction.

In 2009–2010, four independent genome-wide association studies reported SNPs, in the IL28B (IFN-λ3) region, associated with response to treatment (57–60). All patients tested received the SOC (pegylated interferon plus ribavirin). Interestingly, different ethnicities (European, African American, Australian and Japanese) have been included and compared in these three studies.

Ge *et al.* (57) analysed 1137 patients with HCV genotype 1 infection, and they identified several SNPs near the *IL-28B* gene on chromosome 19 that were significantly more common in responders than in nonresponders. A study by Thomas *et al.* (61) reports that the same IL-28B variant, described by Ge and colleagues, is also associated with a spontaneous clearance of HCV.

A strong association of rs12979860 with both EVR and SVR in IFN-naive patients treated with Peg-IFNα-2a/ribavirin was also reported (62). These results extend previous findings to show EVR and SVR associations in patients treated with Peg-IFNα-2a monotherapy and with conventional IFN/ribavirin. Additionally, we rank all previously described SNPs and find that rs12979860 drives the association with response. Finally, we highlight the association of rs12979860 with early HCV decline in response to IFN treatment.

IFN-λs, including IFN-λ1, 2 and 3 (also known as IL-29, IL-28A and IL-28B), are a newly described group of antiviral cytokines distantly related to type I IFNs and IL-10 family members (63,64;). The IFN-λ receptor complex consists of a unique ligand-binding chain, IFN-λR1 (also designated IL-28Rλ), and an accessory chain, IL-10R2, which is shared with receptors for IL-10-related cytokines. IFN-λs binding to its receptor activates pathways of JAK-STATs and MAPKs and induces antiviral, antiproliferative, antitumour and immune responses. IFN-λ proteins seem to have a lower antiviral activity than IFN-α*in vitro* (63). IFN-λ1 has been shown to exhibit dose- and time-dependent HCV inhibition, to increase ISGs expression, and to enhance the antiviral efficacy of IFN-α (64).

Although all of the identified variants in the three studies lie in or near the *IL-28B* gene, none of them has an obvious effect on the function of this gene.

Furthermore, genetic variants leading to inosine triphosphatase deficiency, a condition not thought to be clinically important, protect against haemolytic anaemia in hepatitis-C-infected patients receiving RBV. Two ITPA polymorphisms known to be responsible for inosine triphosphatase deficiency cosegregate with the rs6051702 C allele that strongly associates with protection against haemoglobin reduction in European Americans (65).

### Transcript gene expression

Liver gene expression has been used to determine response to the treatment. Differential expression of genes directly and indirectly implicated in the mechanism of response to PEG-IFN and ribavirin can explain the variation of the treatment efficiency. Gene expression analyses in liver biopsies have been assessed by real-time polymerase chain reaction or microarray studies. Gene expression in responders and non-responders has been compared, in a study, it reported to ISGs upregulated in non-responder patients (66). This observation suggests a possible rationale for treatment resistance.

The expression profile of a selection of genes related to liver dysregulated during HCV infection has been analyzed according to the response to the treatment. A two-gene signature has been successfully identified, IFI27 and CXLC9 (67). This signature predicts the response to the treatment in 79% of the patients, with a predictive accurancy of 100% in non-responders and 70% in sustained virological responders (67). The results also suggest that ISGs are upregulated in non-responders before treatment.

Gene expression analysis in peripheral blood mononuclear cells are actually lacking for HCV. Analyzing genes expression in PMBC rather than in liver biopsie represents an insight for patients because it does not need any invasive exploration. Many of the genes found to be upregulated between non-responders and responders encode molecules secreted in the serum (cytokines) (68, 69). Thus, they could be used in the development of serums markers as predictors of response to HCV treatment.

In a recent study, authors demonstrated that an early expression of interferon-dependent genes can help to predict response rates to PEG-IFN plus ribavirin treatment. Blood samples of 68 patients were collected and results showed that SVR could be predicted by the gene expression of the signal transducer and activator of transcription-6 (STAT-6) and suppressor of cytokine signalling-1 in pretreatment samples. Interestingly, even after 24 h of treatment, interferon-dependent genes expression can help to predict the probability of achieving an SVR (70) (Table 1).

### Serum studies

One study investigated the binding to the CXC chemokine receptor 3 (CXCR3). Interestingly, they demonstrated that the binding of CXCL 10 and CXCL9 decrease during successful anti-HCV treatment, while CXCL11 level is not significantly modified (68). Those results are very promising and these data should be validated in large cohorts before being used as predictors of SVR during treatment.

In a proteomic study of our group, the authors have compared serum proteins expression of 96 patients with chronic hepatitis C (71). Using logistic regression to predict response to the treatment (PEG-IFN plus ribavirin in 89% of all patients), two protein peaks have been identified. Moreover, this algorithm had been used to predict the response to PEG-IFN plus ribavirin in an independent group of patients. The response was predicted correctly in 81% of the patients (71) (Table 1).

## Interaction of hepatitis C virus with interferon pathway

Hepatitis C virus infection induced the IFNβ pathway. During its replication, some dsRNA are generated, and they represent signals that induce the IFN pathway through binding to receptor such as TLR3. Thus, several transcription factors are recruited to activate the transcription of IFNβ.

Some proteins of HCV have been well described for their interaction with the IFN pathway. NS3-4A shows two major anti IFN activities: (i) NS3-4A mediates the cleavage of the C-terminal region of IPS-1/cardif, causing the disruption of NF-kB and IRF-3 activation, probably due to mislocalization of cleaved IPS1/cardif from the mitochondria and (ii) NS3-4A also mediates TRIF proteolysis (11). Thus, HCV proteins may block both TLR and RIG-I-Mad5-dependent signalling pathways to antagonize type I IFN induction. The NS3-4A is a dual therapeutic target whose inhibition may block viral replication and restore IRF-3-dependent control of HCV infection.

Some HCV-related effect may also contribute to attenuation of the IFN pathway. It has been reported that the HCV core protein induce the activation of the suppressor of cytokine signalling 3 (SOCS3) (72). SOCS proteins are known to inhibit cytokine via the JAK/STAT pathway. HCV proteins expression is associated with the accumulation of the proteins phosphatase 2A. Patients with chronic hepatitis C present higher levels of PP2A expression in liver tissue (73). It has been reported that the methylase PRMT1 targets methylation of NS3 on arginine 1493 (in the polyprotein), thus leading to the inhibition of its helicase activity (74). PP2A inhibits PRMT1 methylase activity. It is thought that accumulation of PP2A in patients with chronic hepatitis C, through inhibition of PRMT1, maintains NS3 helicase activity to stimulate HCV replication (74).

A recent study assessed the efficiency of antipolymerase and antiprotease to overcome the inhibitory effect of viral protein expression on the IFN signalling pathway. Interestingly, while antipolymerase does not revert the inhibitory effect of the polymerase, the two antiprotease used counteract NS3/4A protease inhibition, but only in a concentration far in excess (75).

## MicroRNAs and hepatitis C virus infection

MicroRNAs (miRNAs) are a class of small non-coding RNA molecule of 20–22 nucleotides that control gene expression by targeting mRNAs for transcriptional repression or cleavage. They are involved in the regulation of crucial cellular mechanisms such as development, cell differentiation, proliferation and apoptosis.

The importance of the miRNAs machinery in HCV replication has been recently described in various studies. For example, silencing of the RNAse III Dicer by siRNA inhibits HCV replication by about seven-fold (76). miRNA 122 represents 70% of the miRNA expressed in the liver tissue. Moreover, depletion of miRNA 122 in Huh7 hepatoma cells is associated with inhibition of HCV replication and infectious viral production (77). It is thought that miR-122 binding stabilizes the association of HCV mRNA with the ribosome stimulating the translation (78).

These findings suggest that HCV takes advantage of the presence of miR-122 in hepatocytes, which may be a target for novel approaches in the treatment of HCV infection. Interestingly, IFNβ was found to rapidly modulate the expression of numerous cellular miRNAs *in vitro*, and eight of these IFN- β-induced miRNAs were shown to have sequence-predicted targets within the HCV genomic RNA (79). Moreover, IFN-β results in a significant reduction in miR-122 expression. These findings strongly support the notion that the human organism uses cellular miRNAs to overcome HCV infection through the IFN system, and also adds a new component to the antiviral arsenal of IFNs.

However, according to a recent report, it appears that there is no correlation of miR122 expression in the liver with viral load. Moreover the author showed that non-responders present significantly decreased loads of miR122 expression before any treatment (80).

In 2010, Lanford *et al.* (81) demonstrated that treatment of chronically infected chimpanzees with a locked nucleic acid-modified oligonucleotide (SPC3649) complementary to miR-122 leads to long-lasting suppression of HCV viraemia, with no evidence of viral resistance or side effects in the treated animals. Furthermore, transcriptome and histological analysis of liver biopsies demonstrated derepression of target mRNAs with miR-122 seed sites, downregulation of interferon-regulated genes and improvement of HCV-induced liver pathology. The prolonged virological response to SPC3649 treatment without HCV rebound holds promise of a new antiviral therapy with a high barrier to resistance (81).

In 2009, the miRNA 199a has been reported to be a strong regulator of HCV infection. Binding of miRNA 199a targets HCV genome degradation and limits viral replication (82). The authors used a miRNA target search algorithm and identified miRNA 199a that targets the HCV genome. They demonstrated that specific miRNA effectively acts as an RNA silencing-based antiviral response during HCV replication with miRNA-specific machinery. The present study suggests that miRNA mediated HCV inhibition and it may be possible to apply it for the development of novel anti-HCV therapies.

## How to overcome non-response?

There are two major ways to achieve higher rates of SVR: (i) increase the efficiency of the current treatment by modifying the factors of non-response such as obesity, alcohol and drug consumption and later by adding STAT-C to the SOC and (ii) detect non-responders before beginning any treatment.

Several studies have demonstrated that some ISGs are upregulated in non-responders before the treatment (67). Combining those results to genotype and fibrosis score will give strong indications about the chance of each patient to respond to the treatment.

Because of adverse effects, it is important to be able to limit as much as possible treatment that will fail.

On the other hand, new drug therapies such as antiviral protease and antipolymerase are under development. The protease inhibitor NS3/4A telaprevir is being developed by the companies Vertex (Cambridge, MA, USA) and Tibotec (Mechelen, Belgium). The results of the phase II trial have been recently published (83, 84). In these studies, the anti protease has been combined to PEG-IFN +/− ribavirin. Adding telaprevir to the SOC presents two significant advantages: (i) it increases SVR rates from approximately 50 to 70% in patients with genotype 1 and (ii) the duration of the treatment can be reduced from 48 to 24 weeks. Response rates were lowest with the regimen that did not include ribavirin. However, telaprevir regimen is related to higher discontinuations because of adverse events (mainly cutaneous rash) (83, 84).

Boceprevir (Schering Plough, Kenilworth, NJ, USA) is a small molecule, which is a specific inhibitor of the viral protease NS3/4A. After 14 days of monotherapy at 400 mg, three times/day, the viral load was significantly reduced by approximately 1.5 log in non-responders to PEG-IFN. Moreover, the association of PEG-IFN 2b with boceprevir resulted in a marked reduction in viral load of approximately 2.5 log after 13 days of treatment in non-responders to PEG-IFN +/− ribavirin (85).

In genotype 1 patients, when boceprevir is combined with SOC, it appears safe for use up to 48 weeks and substantially improves SVR rates with 28 weeks of therapy. In this trial, 48 weeks of boceprevir treatment almost doubles the SVR compared with the current SOC (86). A 4-week lead-in with SOC before the addition of boceprevir appeared to reduce the incidence of viral breakthrough.

In HCV-genotype 1 infected patients in whom initial peginterferon α and ribavirin treatment failed, retreatment with telaprevir in combination with pegiinterferon α-2a and ribavirin (approximately 50%) was more effective than retreatment with peginterferon α-2a and ribavirin alone (approximately 15%) (87). Polymerase inhibitors interfere with viral replication by binding to the NS5B RNA-dependent RNA polymerase. Two types of polymerase inhibitors are actually under development: (i) nucleoside analogue and (ii) non-nucleosides inhibitors. Nucleoside analogues act as chain terminators; they interfere with the initiation of RNA transcription and elongation. In contrast to the nucleoside analogues that target the active site of HCV polymerase, non-nucleoside inhibitors have been designed to bind to several discrete sites on HCV polymerase. The resistance profiles of nucleoside analogues, non-nucleoside inhibitors and protease inhibitors are all distinct from each other. Thus, it is possible that agents from different classes will act in a complementary manner to increase their efficiency and prevent the development of resistance.

The future of chronic hepatitis C therapy seems to be in the combination of these different drugs with different sites of action (protease and polymerase inhibitors), maybe even in interferon-free regimens. The potential advantages of these combinations should be additive or synergistic antiviral effects, and decrease the occurrence of viral resistance. Unfortunately, so far, few data are available. With the availability of the resistance profile of the future molecules, we should be able to avoid cross resistance.

## Conclusion

Interferon responsiveness remains a major clinical problem in the eradication of HCV, even with the use of new drugs such as protease and polymerase inhibitors.

Some factors associated with low response rates cannot be modified such as male sex, ethnicity, age and genotype, although other factors such as steatosis, drug and alcohol consumption, insulin resistance, and obesity can be controlled and chances of a patient to achieve an SVR could increase.

It has been shown that in non-responders, some ISGs were highly expressed; thus, preactivation of the IFN system in patients appears to limit the effect of IFN antiviral therapy.

Moreover, HCV proteins encode some activities to interfere and limit the IFN signalling pathway. The next step of anti-HCV therapy would probably consist in two major advances: first, in the use of new antiviral drugs such as protease and polymerase inhibitors or drugs that help restore the IFN signalling pathway, and second, the detection of non-responders based on several factors including ISG expression. It would be important to identify genetic and molecular markers that might predict response. However, such markers need to be sensitive and specific, feasible and easy to assess. Protease inhibitors will be registered for genotype 1 patients since they are specific for genotype 1 and not for genotype 2, 3 or 4. The direct antiviral effect of new molecules could reverse IFN resistance. The hope for the next future is that new therapeutic strategies would overcome non-response.

## Abbreviations

PEG-IFN: pegylated interferon
RVR: rapid virological response
SOC: standard of care
STAT-C: specifically targets agents against hepatitis C
SVR: sustained virological response.
